# Clinical, Electroencephalogram and Imaging Characteristics of Patients With Anti‐LGI1 Antibody Encephalitis: A Multicenter Cohort Study

**DOI:** 10.1111/cns.70414

**Published:** 2025-05-05

**Authors:** Yang Zhao, Yue Yuan, Rong‐Ze Wang, Mei Cui, Shu‐Fen Chen, Ke‐Liang Chen, Meng‐Meng Li, Yu‐Yuan Huang, Hai‐Ning Zhang, Yan Zhang, Meng Zhao, Hui Tian, Li Sun, Jin‐Tai Yu

**Affiliations:** ^1^ Department of Neurology The First Hospital of Jilin University Changchun Jilin Province China; ^2^ Department of Neurology and National Center for Neurological Diseases, Huashan Hospital, State Key Laboratory of Medical Neurobiology and MOE Frontiers Center for Brain Science, Shanghai Medical College Fudan University Shanghai China

**Keywords:** anti‐LGI1 encephalitis, autoimmune disease, behavioral and psychological symptoms, electroencephalogram, rapidly progressing cognitive impairment, seizures

## Abstract

**Objectives:**

To summarize the clinical, electroencephalogram (EEG), and imaging characteristics of patients with anti‐leucine‐rich glioma‐inactivated 1 autoimmune encephalitis (LGI1‐AE) and provide a reference for clinical diagnosis and treatment.

**Methods:**

We retrospectively analyzed 88 patients diagnosed with LGI1‐AE between January 2018 and April 2024 in the Department of Neurology, Huashan Hospital, Fudan University, and the First Hospital of Jilin University.

**Results:**

This retrospective study analyzed 88 patients diagnosed with LGI1‐AE. The initial clinical presentation predominantly featured rapidly progressive cognitive impairment (RPCI) (51.1%) and seizures (50%). Brain magnetic resonance imaging and 18 F‐fluorodeoxyglucose positron emission tomography (18F‐FDG PET) indicated predominant lesion localization in the unilateral or bilateral temporal lobe and/or basal ganglia. Abnormal EEG was observed in 66 cases (79.5%). LGI1‐AE cases had increased power in the low‐frequency bands (δ and θ) compared to normal controls. Low‐frequency band (δ and θ) power in T3 and Fz channels was positively correlated with LGI1 antibody titers in cerebrospinal fluid (CSF). Spearman correlation analysis showed that baseline modified Rankin Scale (mRS) scores were correlated with serum antibody titers and CSF antibody titers.

**Conclusions:**

Baseline mRS scores and low‐frequency power in the frontotemporal region showed a positive correlation with anti‐LGI1 antibody titers, suggesting that antibody levels may reflect disease severity in LGI1 autoimmune encephalitis. Further studies are warranted to validate these associations in prospective multicenter cohorts.

## Graphical abstract

To summarize and analyze the general clinical manifestations, laboratory tests, electroencephalography and imaging characteristics, treatment and prognosis of 88 patients with anti‐leucine‐rich glioma inactivated protein 1 antibody encephalitis (LGI1‐AE) at baseline, discharge, and follow‐up in China, aiming to identify risk factors affecting disease severity and guide clinical practice.

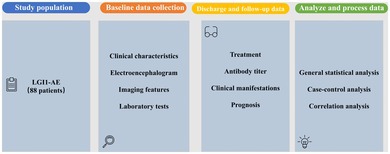

## 1 Introduction

Anti‐leucine‐rich glioma‐inactivated 1 autoimmune encephalitis (LGI1‐AE) is a common type of autoimmune encephalitis [1]. Clinical manifestations typically include rapidly progressive cognitive impairment, mental behavioral abnormalities, seizures, refractory hyponatremia, neuropathy or autonomic dysfunction, and other coexisting peripheral symptoms [2].

The diagnosis of LGI1‐AE is established by detecting anti‐leucine‐rich glioma‐inactivated 1 (anti‐LGI1) antibodies in serum and/or cerebrospinal fluid (CSF). LGI1 is a secreted neuronal protein. Combined immunofluorescence and mass spectrometry analyses have shown that LGI1 is enriched in excitatory and inhibitory synaptic contact sites, most densely located within the CA3 regions of the hippocampus [3]. In recent years, LGI1‐AE has attracted wide attention, and several studies have described its clinical features [4, 5]. Notably, the risk factors influencing disease severity, unfavorable outcomes, and relapse potential in LGI1‐AE remain inadequately characterized. Therefore, this study summarizes and analyzes the clinical manifestations, laboratory tests, electroencephalography (EEG), imaging features, and potential risk factors of 88 patients with LGI1‐AE in two centres in China, aiming to provide clinicians with a deeper understanding of better diagnosis and treatment.

## 2 Methods

### 2.1 Study Protocol Approvals, Registrations, and Patient Consents

This study was approved by the Ethics Committees of Huashan Hospital, Fudan University, and the First Hospital of Jilin University, both of which granted an exemption from obtaining informed consent based on compliance with ethical requirements.

### 2.2 Study Participants

A total of 88 patients with LGI1‐AE who met the diagnostic criteria outlined in the Expert Consensus on the Diagnosis of Autoimmune Encephalitis in China (2022 edition) [6], along with 16 healthy controls, were recruited from the Department of Neurology at Huashan Hospital, Fudan University, and the First Hospital of Jilin University between January 2018 and April 2024.

### 2.3 Data Collection

The detailed clinical data of the patients were analyzed retrospectively, including general data, clinical symptoms and signs, neuropsychological assessments, laboratory examinations, brain magnetic resonance imaging (MRI), EEG, 18F‐fluorodeoxyglucose positron emission tomography (18F‐FDG PET), treatment, and follow‐up and prognosis assessments.

### 2.4 Clinical Evaluations

All patients were examined for autoimmune encephalitis‐associated antibody spectrum and paraneoplastic antibody spectrum in serum or CSF. The antibody titers are classified into the following grades: 0 (negative), grade 1 (< 1:10), grade 2 (< 1:100), grade 3 (≥ 1:100), and grade 4 (≥ 1:320). The modified Rankin scale (mRS) and Montreal Cognitive Assessment (MoCA) [7] were used to evaluate the neurological and cognitive status of the patients. All discharged patients were followed up by telephone or outpatient until now. Relapse is defined as the recurrence of new symptoms or aggravation of the original symptoms more than 2 months after the symptoms have stabilized or improved [8].

### 2.5 EEG Data Collection and Processing

EEG was recorded within five days after diagnosis. Using an American Biologic EEG instrument, 19 Ag/AgCl electrodes were fixed on elastic caps according to the 10–20 international system (reference CPz) and grounded in the AFz to record EEG. The 19 recording electrodes were: Fp1, Fp2, F3, F4, C3, C4, P3, P4, O1, O2, F7, F8, T3, T4, T5, T6, Fz, Cz, Pz. EEG seizures were recorded without significant noise, and various artifacts (including motor artifacts, eye movements, and blinking) were captured under visual examination by two experienced physicians and technicians.

EEG data was imported into Python, and the MNE library was used for offline processing [9]. First, the recorded EEG raw data was preprocessed: useless electrodes were removed, and the whole brain electrode average was used for re‐referencing. The filtering range was set to 1–30 Hz, with a sampling rate of 500 Hz. Components related to eye movement and muscle activity were removed using independent component analysis (ICA). Subsequently, Welch's method [10] was employed for spectrum analysis, calculating the absolute power and power spectral density (PSD) for the frequency bands: δ (2–4 Hz), θ (4–8 Hz), α1 (8–11 Hz), α2 (11–13 Hz), β1 (13–20 Hz), and β2 (20–30 Hz). The ratio of (δ + θ)/(α + β) for each electrode, referred to as the DTABR, was also computed. Finally, the drawing function provided by the MNE library was used to visualize the calculated PSD results.

### 2.6 Statistical Analysis

Statistical analyses were conducted using Microsoft Excel and SPSS Statistics 27.0, while Origin 2021 was utilized for data visualization. We used the Shapiro–Wilk test to assess data normality. Normally distributed continuous variables were expressed as mean ± standard deviation (*x̄* ± SD), whereas non‐normally distributed data were presented as median (interquartile range) [M (P25, P75)]. To compare quantitative EEG characteristics between the healthy control group and LGI1‐AE patients, we applied either the independent samples *t*‐test (for normally distributed data) or the Mann–Whitney *U* test (for non‐normally distributed data). Correlation analyses were performed using Pearson's test (for normally distributed variables) or Spearman's rank correlation (for non‐normally distributed variables), depending on data distribution. These methods were used to assess relationships between serum/CSF titers and other test results. All statistical tests were two‐tailed, with a *p*‐value < 0.05 considered statistically significant. The study protocol and statistical analysis plan are available in eSAP 1.

## 3 Result

### 3.1 Clinical Characteristics

Among the 88 cases, 53.4% were male. The mean age at onset was 58.99 ± 12.30 years, with a median disease duration of 60 days (interquartile interval: 30–120 days). Initial clinical manifestations predominantly included rapidly progressive cognitive impairment (RPCI) (51.1%) and seizures (50%). During disease progression, 81.8% of cases developed epileptic seizures, comprising 46 cases of generalized tonic–clonic seizures (GTCS). Notably, 36 cases exhibited faciobrachial dystonic seizures (FBDS), and 8 cases demonstrated transient global amnesia (TGA). Baseline Montreal Cognitive Assessment (MoCA) scores were available for 40 patients (45.5%), yielding a mean score of 15.3 ± 5.6 (Table 1). Among the eight cognitive domains evaluated by the scale, the recall domain exhibited the most severe impairment.

**TABLE 1:** Baseline MoCA cognitive subdomain scores in LGI1‐AE.

Items	Total points	Mean values
MoCA	30	15.3 ± 5.6
Cognitive domain		
Visuospatial and executive ability	5	2.4 ± 1.2
Naming	3	1.9 ± 1
Attention and concentration	3	2.3 ± 0.9
Computation	3	2 ± 0.9
Verbal fluency	3	1.3 ± 1
Abstraction	2	0.5 ± 0.6
Recall	5	0.6 ± 1
Orientation	6	3.6 ± 1.7

Abbreviation: MoCA, montreal cognitive assessment.

RPCI was observed in 64 cases (72.7%), while behavioral and psychological symptoms (BPS) were observed in 42 cases (47.7%), primarily characterized by gibberish, behavioral anomalies, hallucinations, and personality changes. Sleep disorders were present in 32 cases (36.4%), including rapid eye movement sleep behavior disorder (RBD) in 23 cases and insomnia in 13 cases. Additionally, 24 cases (27.3%) showed an altered level of consciousness. 10 cases combined with other symptoms, including 4 cases with autonomic symptoms, mainly manifested as episodes of profuse sweating, goose bumps, flushing, and nausea. 4 cases had aphasia or speech clumsiness, 1 case reported a sensation of electric shock in the body, and 1 case had generalized pain. All cases were screened for tumors, with 7 cases detected, including peripheral lung cancer, prostate cancer, thymoma, gastrointestinal tract tumors, breast papilloma, uterine fibroids, and acute leukemia (Table 2).

**TABLE 2:** Demographics and clinical presentations in LGI1‐AE.

Items	Values
Gender (male/female)	(47/41)
Age of onset [year, mean (SD)]	58.99 (12.30)
Duration of onset [day, M (P25, P75)]	60 (30–120)
Initial symptom (*n*%)	
RPCI	45 (51.1%)
BPS	23 (26.1%)
Consciousness disorder	10 (11.4%)
Epilepsy	44 (50%)
Sleep disorders	9 (10.2%)
Others	3 (3.4%)
Clinical symptoms (*n*%)	
RPCI	64 (72.7%)
BPS	42 (47.7%)
Consciousness disorder	24 (27.3%)
Epilepsy	72 (81.8%)
GTCS	46
FES	41
FBDS	36
TGA	8
Sleep disorders	32 (36.4%)
Agrypnia	13
RBD	23
Others	10 (11.4%)
Associated with tumor (*n*%)	7 (8.0%)

Abbreviations: BPS, behavioral and psychological symptoms; FBDS, faciobrachial dystonic seizure; FES, focal seizures; GTCS, Generalized tonic–clonic seizure; RBD, rapid eye movement sleep behavior disorder; RPCI, rapidly progressing cognitive impairment; TGA, transient global amnesia.

### 3.2 Laboratory Examinations

A total of 77 cases underwent lumbar puncture during hospitalization, during which CSF pressure, routine tests, biochemistry, and cytology were analyzed. The mean pressure was 137.24 ± 5.25 mmH_2_O (normal range: 80–180 mmH_2_O). The median leukocyte count was 3 × 10^6^/L (interquartile range: 1–4.75 × 10^6^/L) (normal range: 0–5 × 10^6^/L) the median protein was 0.409 g/L (interquartile range: 0.313–0.514 g/L) (normal range: 0.15–0.45 g/L), and the mean chloride value was 120.55 ± 0.64 mmol/L (normal range: 120–130 mmol/L).

A total of 77 cases underwent testing for the autoimmune encephalitis antibody spectrum and the paraneoplastic syndrome antibody spectrum in both serum and CSF. Among these, all 77 (100%) cases were serum anti‐LGI1 antibodies positive, 73 (96.1%) cases were both serum and CSF anti‐LGI1 antibodies positive, 4 cases were serum positive while CSF negative, and 4 cases were complicated with other antibodies (anti‐mGluR5 antibody, anti‐Amphiphysin antibody, anti‐Titin antibody, and anti‐Yo antibody). Hyponatremia was present in 51 of 88 cases (58%) (Table 3).

**TABLE 3:** Laboratory and imaging characteristics in LGI1‐AE.

Items	Values
Abnormal serum tumor markers (*n*%)	6 (6.8%)
Serum sodium	51 (58%)
Intracranial pressure [mmH_2_O, mean (SD)]	137.24 (5.25)
CSF^a^	
WBC [×10^6^/L, M (P25, P75)]	3 (1–4.75)
Protein [g/L, M (P25, P75)]	0.409 (0.313–0.514)
Chloride [mmol/L, mean (SD)]	120.55 (0.64)
Positive serum anti‐LGI1‐Ab^b^	77 (100%)
Positive CSF anti‐LGI1‐Ab	73 (96.1%)
Codetection with other AEAb	5
MRI^c^	39 (49.4%)
Temporal lobe	37 (45.8%)
Unilateral involved	14
Bilateral involved	23
Basal ganglia	6 (7.6%)
Unilateral involved	3
Bilateral involved	3
Other lobes	13 (16.5%)
18F‐FDG PET^d^	16 (72.7%)
Increase intake in temporal lobe	13 (59.1%)
Unilateral involved	4
Bilateral involved	9
Increase intake in basal ganglia	5 (22.7%)
Increase intake in other lobes	1 (4.5%)
Abnormal EEG^e^	66 (79.5%)
Background activity	37 (44.6%)
Clinical seizures	11 (13.3%)
Subclinical seizures	28 (34.1%)
Interparoxysmal period	39 (47.6%)

Abbreviations: 18F‐FDG PET, 18 F‐fluorodeoxyglucose positron emission tomography; AEAb, autoimmune encephalitis antibody; anti‐LGI1‐Ab, anti‐leucine‐rich glioma‐inactivated 1 antibody; CSF, cerebrospinal fluid; EEG, electroencephalogram; MRI, magnetic resonance imaging; WBC, white blood cell.

^a^ 76 cases were sent for CSF anti‐LGI1‐Ab.

^b^ 77 cases were sent for serum anti‐LGI1‐Ab.

^c^ 79 cases underwent brain MRI.

^d^ 22 cases underwent 18F‐FDG PET.

^e^ 82 cases underwent EEG.

Spearman correlation analysis revealed that baseline mRS was correlated with age of onset (*ρ* = 0.280, *p* < 0.01), MoCA score (*ρ* = −0.353, *p* < 0.05), serum antibody titers (*ρ* = 0.345, *p* < 0.01) and CSF antibody titers (*ρ* = 0.479, *p* < 0.001). In contrast, no correlations were found between recurrence and general demographic data or laboratory examinations. Notably, correlations were observed between serum antibody titers and gender (*ρ* = 0.272, *p* < 0.05), CSF white blood cell (WBC) counts (*ρ* = 0.491, *p* < 0.01) and CSF antibody titers (*ρ* = 0.565, *p* < 0.01). Additionally, there was a correlation between CSF antibody titers and CSF WBC counts (*ρ* = 0.259, *p* < 0.05) (Table 4).

**TABLE 4:** Associations of disability, recurrence, and LGI1 antibody levels with clinical and laboratory markers.

Items	Baseline mRS	Recurrence	Serum anti‐LGI1‐Ab titer^a^	CSF anti‐LGI1‐Ab titer^a^
Gender	0.124	0.092	0.272*	0.166
Age of onset	0.280**	0.102	0.108	0.069
Duration of onset	0.114	−0.143	−0.004	0.027
Serum sodium	−0.195	0.087	−0.137	−0.207
Intracranial pressure	−0.152	−0.056	0.046	−0.221
CSF WBC	0.169	0.125	0.491**	0.259*
CSF Protein	0.032	0.054	−0.126	−0.004
CSF Chloride	−0.092	−0.017	0.043	−0.147
MoCA	−0.353*	0.064	−0.204	−0.138
CSF anti‐LGI1‐Ab titer^a^	0.345**	0.183	0.565**	1
Serum anti‐LGI1‐Ab titer^a^	0.479***	0.065	1	—
Recurrence	0.019	1	—	—

^a^ Serum and cerebrospinal fluid antibody titer data were missing in 3 cases. *Note*: Significance of bold values indicate statistical significance at the specified levels.

*0.01 ≤ *p* < 0.05; **0.001 ≤ *p* < 0.01; ***p < 0.001.

After adjusting for potential confounding factors, including sex, age, and disease duration, the baseline mRS maintained robust correlations with serum antibody titers (partial *ρ* = 0.552, *p* < 0.01) and CSF antibody titers (partial *ρ* = 0.525, *p* < 0.01). These findings suggest that in patients with LGI1‐AE, serum/CSF antibody levels and acute‐phase neurological impairment severity demonstrate interrelationships independent of demographic characteristics and disease features. Similarly, the correlation between serum and CSF antibody titers remained robust (partial *ρ* = 0.561, *p* < 0.01). The attenuated correlation between baseline mRS and MoCA (partial *ρ* = 0.037, *p* > 0.05) alongside the strengthened association between CSF antibody titers and CSF WBC (partial *ρ* = 0.414, *p* < 0.05) after adjustments highlights that weak correlations necessitate rigorous confounder adjustment. This pattern suggests that strengthened associations may indicate true pathophysiological linkages, whereas weakened relationships likely reflect confounding interference (Table S1).

### 3.3 Imaging Examinations

**FIGURE 1:**
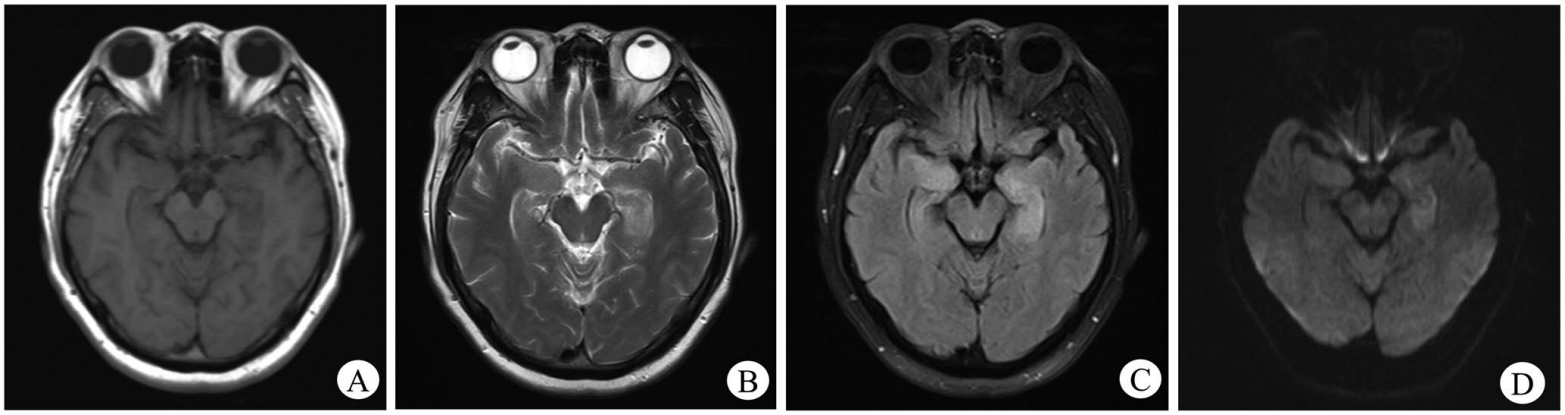
Bilateral hippocampal abnormalities on MRI in LGI1‐AE. Irregular patchy abnormal signals are seen bilaterally in the hippocampal region of the deep temporal lobes, with slightly low signals in T1WI (A) and slightly high signals in T2WI (B), T2WI‐FLAIR (C) and DWI (D).

**FIGURE 2:**
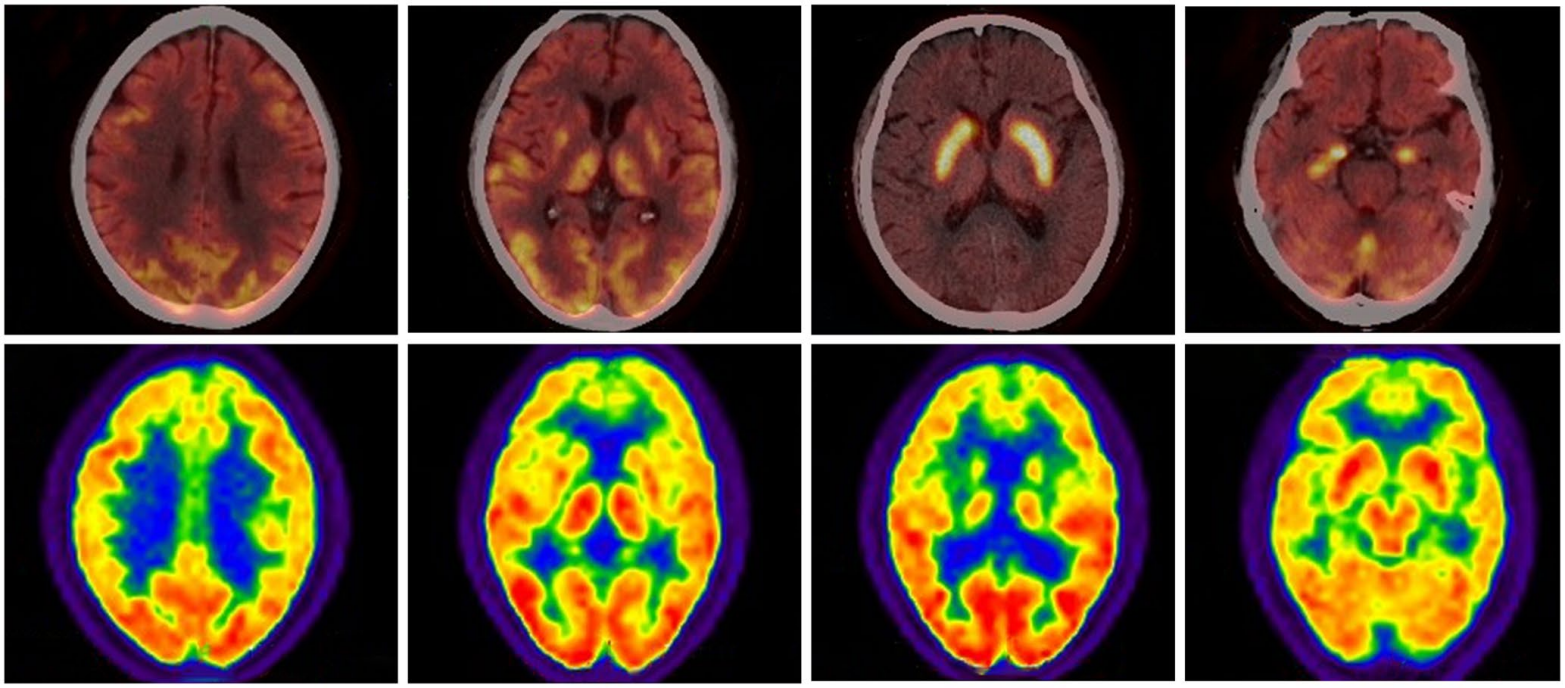
Bilateral temporal hypermetabolism on 18F‐FDG PET in LGI1‐AE. 18F‐FDG PET demonstrates bilateral hypermetabolism in the deep temporal lobes adjacent to the hippocampus.

A total of 79 cases underwent brain MRI, of which 39 cases (49.4%) exhibited abnormalities (Figure 1). Among these, 37 cases (45.8%) demonstrated temporal lobe involvement, of which 23 were bilateral and 14 were unilateral. The basal ganglia region was involved in 6 (7.6%) cases, of which 3 were bilateral and 3 were unilateral. Other cerebral lobes (frontal, parietal, etc.) were involved in 13 cases (16.5%). 22 cases underwent 18F‐FDG PET examination, and 16 (72.7%) were abnormal (Figure 2). 13 cases (59.1%) showed high uptake in the temporal lobe, including 9 cases with bilateral involvement, 4 cases with unilateral involvement, 5 (22.7%) cases showed high uptake in basal ganglia, and 1 (4.5%) case showed uneven increase of fluorodeoxyglucose (FDG) metabolism in the right cerebellum and the right parietal occipital lobe (Table 3).

### 3.4 EEG

A total of 82 cases underwent EEG examination, with 66 cases (79.5%) exhibiting abnormal EEG findings. Among these, 37 cases (44.6%) demonstrated abnormal background activity, characterized by the disappearance or irregularity of the α rhythm, instability, poor regulation and amplitude modulation, slow frequency, bilateral hemispherical asymmetry, and increased β, θ or δ activity, along with changes in the sleep cycle. During the examination, 11 cases (13.3%) exhibited clinical seizures, presenting with manifestations such as FDBS, RBD, daze, stupor, and automatism. Synchronous electroencephalography usually manifested as extensive high‐amplitude sharp, slow, and sharp‐slow compound wave releases. 28 cases (34.1%) had subclinical seizures, characterized by focal onset electrical seizures in the parietal, occipital, and posterior temporal regions, which lasted 10s–150s to recover. There were 39 cases (47.6%) with abnormal interparoxysmal periods, which showed regular or irregular sharp waves or sharp slow compound waves emitted synchronously or asynchronously in frontal and temporal areas (Table 3).

### 3.5 Power Spectrum Analysis Between LGI‐AE Group and NC Group at Baseline

**FIGURE 3:**
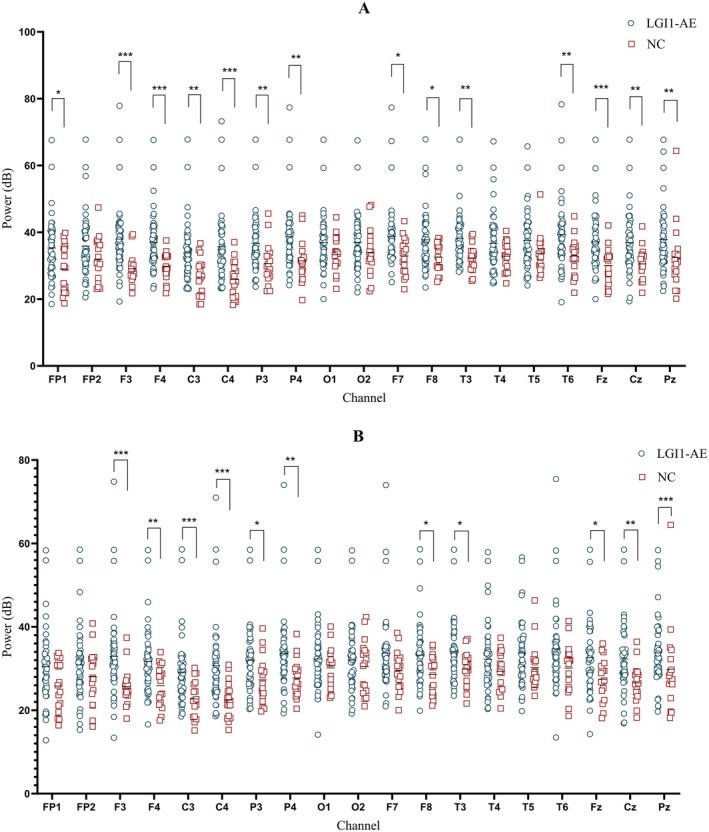
Power values of each frequency band in the LGI1‐AE group and NC group. (A) Comparison of δ‐band power values between the LGI1‐AE group and NC group. (B) Comparison of θ‐band power values between the LGI1‐AE group and NC group. *0.01 ≤ *p* < 0.05; **0.001 ≤ *p* < 0.01; ***p < 0.001. LGI1‐AE, LGI1 antibody‐associated autoimmune encephalitis; NC, normal control.

**FIGURE 4:**
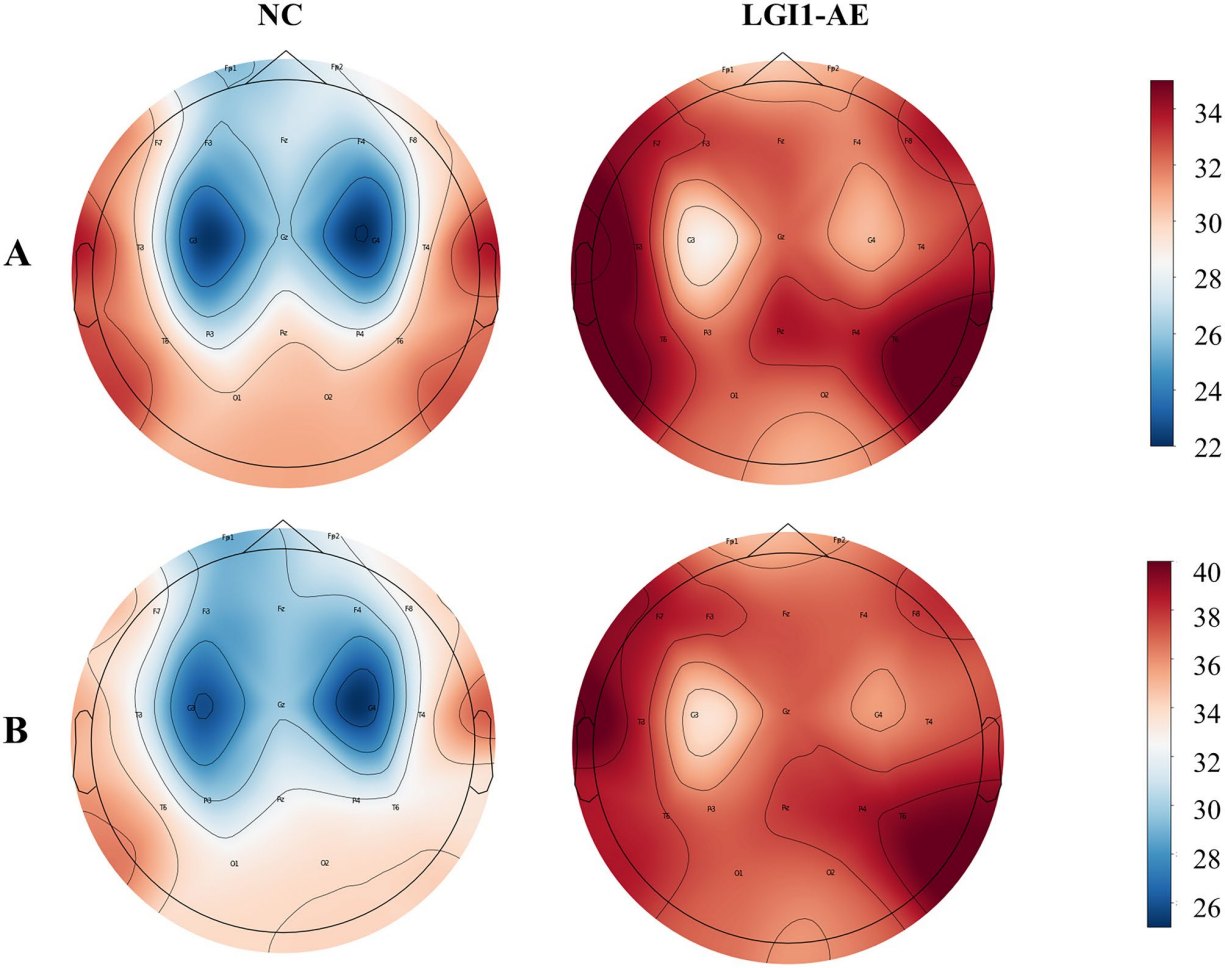
Visualization of PSD results in the LGI1‐AE group and NC group. (A) PSD distribution of NC group and LGI1‐AE group in the θ band. (B) PSD distribution of NC group and LGI1‐AE group in the δ band. PSD, power spectral density.

**FIGURE 5:**
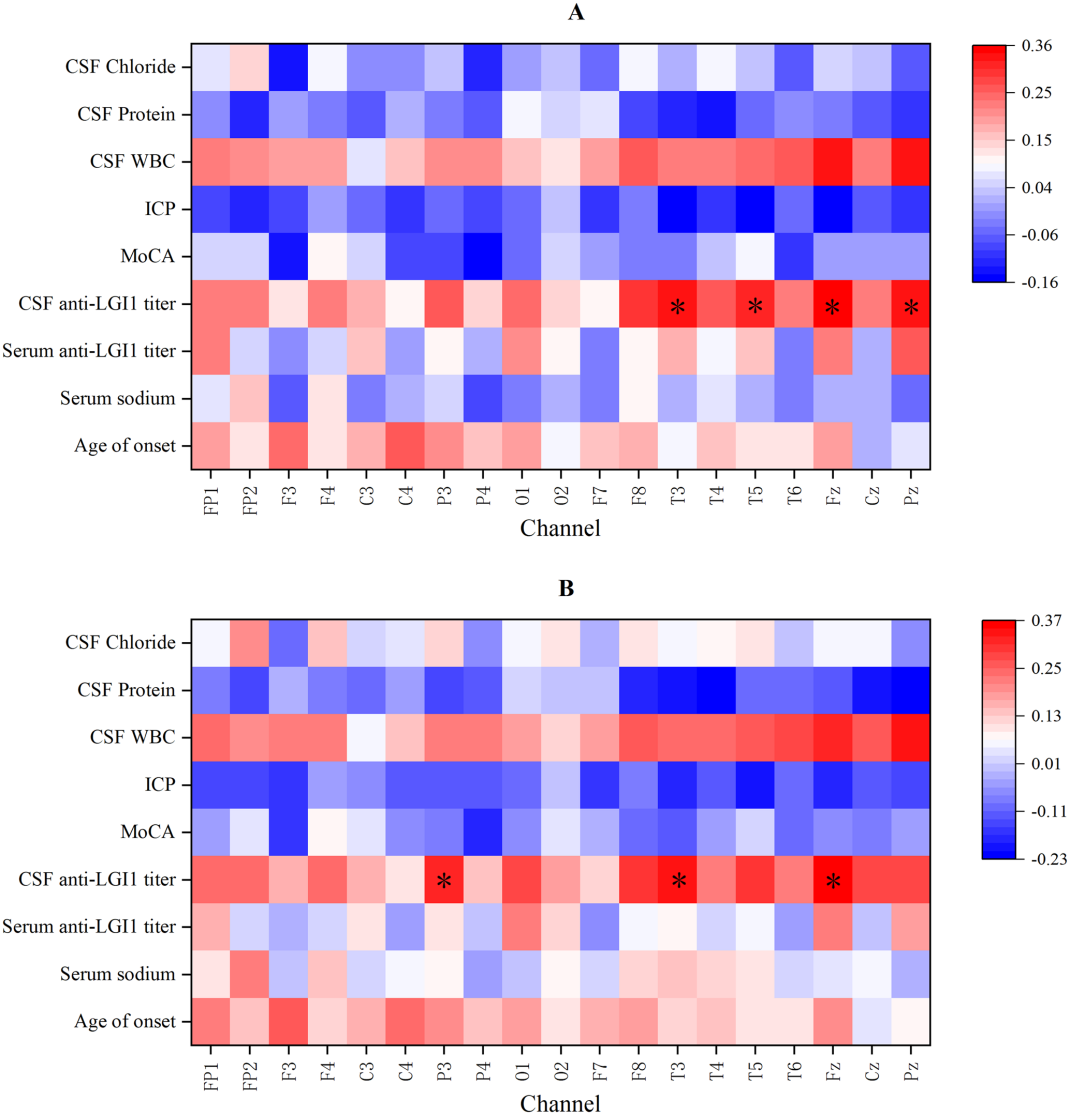
Correlation analysis of 19‐channel spectral power and clinical indicators. (A) Spearman correlation analysis plot of the δ‐band power versus other metrics across 19 channels. (B) Spearman correlation analysis plot of the θ‐band power versus other metrics across 19 channels. *0.01 ≤ *p* < 0.05; **0.001 ≤ *p* < 0.01;***p < 0.001. Anti‐LGI1 titer, anti‐LGI1 antibody titer; ICP, intracranial pressure.

In this study, we randomly selected 47 cases with LGI‐AE at baseline and 17 healthy controls for quantitative EEG analysis. There was no significant difference in age and sex between the two groups (Table S2). First, we compared the average power values of each electrode across different frequency bands. The δ and θ bands power at F3, F4, C3, C4, P3, P4, F8, T3, Fz, Cz, and Pz channels (*p* < 0.05) in the LGI1‐AE group were significantly higher than those in the control group, suggesting that the brain function of the corresponding regions in the LGI1‐AE group was impaired (Figure 3, Figures S1–S4). In the two groups, the PSD distribution maps of θ and δ bands showed that the differences were mainly in the frontal, central, parietal, and some areas of temporal regions (Figure 4). Spearman correlation analysis revealed that δ band power at the T3, T5, Fz, and Pz channels (*p* < 0.05) and θ band power at the P3, T3, and Fz channels (*p* < 0.05) were positively correlated with the CSF LGI1 antibody titers (Figure 5).

### 3.6 Treatment and Prognosis

All 88 cases (100%) were treated with first‐line immunotherapy, including intravenous immunoglobulin (IVIg), intravenous methylprednisolone (IVMP) or plasma exchange (PE). 40 cases (45.5%) received IVMP alone, and 33 cases (37.5%) received both IVMP and IVIg. 15 cases (17%) underwent PE. Due to poor response to first‐line immunotherapy, one patient was treated with rituximab. Due to disease progression, 19 cases (21.6%) received immunosuppressants (azathioprine or mycophenolate mofetil). Before discharge, RPCI, epilepsy, BPS, sleep disorders, and other symptoms (speech disorders, autonomic symptoms, etc.) showed improvement rates of 46.9%, 79.2%, 78.6%, 81.3%, and 70%, respectively (Table 5).

**TABLE 5:** Treatment and follow‐up in LGI1‐AE.

Items	Values
Treatment (*n*%)	
First‐line immunotherapy	
High dose glucocorticoid therapy	40 (45.5%)
High dose glucocorticoid therapy + IvIg	33 (37.5%)
High dose glucocorticoid therapy + PE	15 (17%)
Second‐line immunotherapy	
Rituximab	1 (1.1%)
Long term immunosuppressant therapy	19 (21.6%)
Mycophenolate mofetil	18 (20.5%)
Azathioprine	1 (1.1%)
Symptom improve before discharge (*n*%)	
RPCI	30 (46.9%)
BPS	33 (78.6%)
Epilepsy	57 (79.2%)
Sleep disorders	26 (81.3%)
Others	7 (70%)
Follow up (*n*%)	
Follow up percent	75 (85.2%)
With mild ecmnesia	16 (21.3%)
With well controlled epilepsy	6 (8%)
Residual autonomic dysfunction	2 (2.7%)
Positive antibody titer on retest	5 (7.9%)^a^
Improvement without relapse and continued antibody negativity	41 (65.1%)^a^
Improvement without relapse but persistently antibody‐positive	4 (6.3%)^a^
Number of recurrence	17 (22.7%)

Abbreviations: BPS, behavioral and psychological symptoms; IvIg, intravenous immunoglobulin; PE, plasma exchange; RPCI, rapidly progressive cognitive impairment.

^a^ Blood or cerebrospinal fluid antibodies were not reexamined in 12 cases.

After immunotherapy, 75 cases (85.2%) were followed up, including 18 patients for 1 year, 14 for 2 years, and 43 for more than 2 years. Among them, 16 (21.3%) had mild memory impairment, 6 (8%) had controlled epilepsy, and 2 (2.7%) had residual autonomic dysfunction (e.g., goosebumps, flushed face). 17 cases (22.7%) relapsed, 16 recurred within 1 year, and 1 recurred after 1 year. Of these, 13 (76.5%) had seizures, 7 (41.2%) had BPS, 5 (29.4%) had MCI, and 3 (17.7%) had sleep disorders (Table 5). Antibody titers were rechecked within 2 years after the end of therapy in 63 patients, of which 5 cases (7.9%) had decreased antibody titers but remained positive, 41 cases (65.1%) had improved symptoms but did not recur and remained negative for antibodies, and 4 cases (6.3%) had improved conditions but remained positive for antibodies (Table 5). The efficacy was evaluated by mRS. 72 cases (81.8%) improved after immunotherapy (mRS ≤ 2).

## 4 Discussion

LGI1 antibody (LGI1‐Ab) is an autoantibody targeting leucine‐rich glioma‐inactivated 1 protein. This protein is predominantly localized in the hippocampus and temporal cortex, specifically expressed in neuronal axons and the initial segments of glutamatergic synapses [11]. LGI1‐Ab alters Kv1.1 and α‐amino‐3‐hydroxy‐5‐methyl‐4‐isoxazole‐propionate (AMPA) receptor functions, leading to neuronal hyperexcitability, synaptic plasticity impairment, and reversible memory deficits [12]. LGI1‐Ab‐associated encephalitis represents the second most prevalent form of limbic encephalitis (LE) after anti‐N‐methyl‐D‐aspartate (anti‐NMDA) encephalitis, with its reported incidence rising substantially alongside advancements in detection techniques [13]. This condition predominantly affects middle‐aged and elderly populations, with male predominance. Clinical presentations typically manifest acutely or subacutely [6]. Consistent with prior reports, our cohort demonstrated male predominance (53.4%), a mean onset age of 58.99 ± 12.30 years, and median disease duration of 60 days (interquartile interval: 30–120 days).

While early reports emphasized FBDS as characteristic initial manifestations, other focal attacks in the early stage and memory impairment occurred with the progression of the disease [13]. Our findings reveal RPCI in 51.1% of initial presentations and epileptic seizures (including generalized seizures and FBDS) in 50% of cases. These observations confirm RPCI as a frequent early indicator of LGI1‐AE, underscoring the necessity for clinical suspicion of LGI1 encephalitis in patients presenting with RPCI. In this cohort, 64 cases (72.7%) exhibited RPCI during disease progression. Neuropsychological evaluation using the MoCA revealed the recall domain demonstrated the most pronounced impairment across eight assessed cognitive domains. This characteristic amnestic pattern may correlate with both the predominant hippocampal and temporal cortical distribution of LGI1 protein [14] and the observed focal atrophy in hippocampal CA3 subregions following inflammatory processes. Following immunotherapy, 46.9% of patients achieved cognitive symptom amelioration prior to discharge. Notably, 21.3% retained residual cognitive deficits, indicating chronic cognitive dysfunction represents a frequent sequela in LGI1‐AE.

The BPS of LGI1‐AE patients were mainly manifested as gibberish, behavioral abnormalities, hallucinations, and personality changes, which may be related to the intensive expression of limbic system autoantigens. In this study, 47.7% of the cases had BPS; symptomatic treatment was poor, and most symptoms (78.6%) could be improved rapidly after immunotherapy. LGI1‐AE may also correlate with autonomic dysfunction, though the specific mechanism remains unclear and could involve LGI1‐Ab affecting autonomic synaptic transmission. The main manifestations of autonomic dysfunction in this study were paroxysmal sweating, goosebumps, flushed face, and nausea. Since AE can affect any brain network involved in initiating and regulating sleep [15], all types of sleep disorders may occur, including insomnia, lethargy, RBD, etc. In this study, 32 cases (36.4%) presented with these sleep disorders. Following systemic immunotherapy during hospitalization, 81.3% of cases showed improvement in sleep disturbances by discharge.

Seizures occur in 80%–97% of LGI1‐AE patients, manifesting in diverse forms, including both generalized and localized types. The underlying mechanism involves genetic or acquired loss of the LGI1‐ADAM22 interaction, which reduces AMPA receptor function and contributes to epileptogenesis [16]. FBDS, a hallmark seizure type associated with this encephalitis, has an incidence of 34%–53%. In this study, seizures occurred in 81.8% of cases, with GTCS observed in 52.3% and FBDS in 40.9%, aligning with prior reports. Controversy persists regarding whether FBDS mechanisms originate from epileptic seizures [17].

Shao et al. reported that patients with FBDS exhibited a higher likelihood of basal ganglia (BG) lesions than those without FBDS, strengthening the hypothesis of BG involvement in FBDS pathogenesis [18]. However, Finke et al. found no significant association [19]. In this cohort, 6 of 36 FBDS patients had abnormal MRI signals in the BG. Seizures in LGI1‐AE are often refractory to antiepileptic drugs but respond effectively to immunotherapy, with early intervention preventing symptom progression [20]. This study observed improvement in epileptic symptoms in 79.2% of patients following early immunotherapy.

EEG examination plays a critical role in diagnosing LGI1‐AE, though findings are non‐specific. In this study, 66 of 82 cases (79.5%) exhibited abnormal EEG patterns. Among these, 37 patients (44.6%) showed abnormal background rhythms, which were characterized by disappearance or irregularity of α rhythm, instability, poor regulation and amplitude modulation, slowed frequency, bilateral hemispherical asymmetry, increased β, θ or δ activity, and disrupted sleep architecture. Quantitative EEG analyses revealed significantly elevated power in low‐frequency bands (δ and θ) in the LGI1‐AE group compared to controls, confirming cerebral slowing as a hallmark feature. We propose that these low‐frequency alterations may reflect inflammatory processes, neuronal injury, synaptic dysfunction, and other encephalitis‐related pathophysiological mechanisms. Notably, correlation analyses demonstrated positive associations between low‐band (δ and θ) power at T3 and Fz electrode sites and CSF LGI1 antibody titers (but not serum titers). This observation may align with the neuroanatomical distribution of LGI1, which is predominantly expressed in the temporal lobe and hippocampal cortex, suggesting that antibody‐mediated effects on these regions could modulate low‐frequency activity. These findings support further investigation into low‐band EEG metrics as potential biomarkers for early diagnosis and prognostic evaluation in autoimmune encephalitis.

Hyponatremia represents a frequent clinical feature of LGI1‐AE, observed in 58% of cases in this cohort. This electrolyte imbalance is attributed to dual LGI1 expression in the hypothalamus and kidneys. LGI1‐Ab binds to antidiuretic hormone (ADH), producing neurons in the hypothalamic paraventricular nucleus, inducing excessive ADH secretion, which drives water retention and subsequent hyponatremia. Furthermore, renal tubular expression of LGI1 has been identified, and impaired sodium reabsorption in these tubules exacerbates hyponatremia [14, 21]. Beyond hyponatremia, recent studies by Gadoth et al. report concurrent electrolyte disturbances (e.g., hypomagnesemia, hypophosphatemia) in ≥ 40% of LGI1‐AE patients, suggesting these abnormalities may serve as auxiliary diagnostic indicators [22]. However, the pathophysiological mechanisms underlying these electrolyte imbalances remain undetermined.

In LGI1‐AE, CSF analysis typically lacks distinctive biochemical abnormalities, with cell counts and protein levels often normal or mildly elevated. The detection of LGI1‐Ab in serum or CSF serves as a key diagnostic criterion. Anti‐LGI1‐Ab exert neurotoxic effects, contributing to disease pathogenesis [6]. High‐titer serum antibodies can partially cross the blood–brain barrier (BBB), with serum titers averaging 127‐fold higher than CSF titers [23]. Elevated antibody titers correlate with heightened neurotoxicity, adversely impacting prognosis and relapse risk [8]. Notably, Zhang et al. corroborated that CSF LGI1‐Ab titers closely parallel serum titers, suggesting a peripheral origin of CSF antibodies [24]. CSF‐positive patients exhibited higher rates of psychiatric symptoms, hyponatremia, and poorer outcomes, indicative of more severe neuronal injury. In this study, the serum antibody titers of cases were generally higher than those of CSF‐Ab titers, aligning with prior findings [13]. Acute‐phase antibody titers (serum and CSF) correlated with mRS scores but showed no association with relapse rates. Notably, 4 patients (6.3%) achieved clinical remission without recurrence despite persistent antibody positivity, underscoring that antibody persistence does not invariably predict relapse.

This multivariate analysis elucidates the complex clinical correlations in anti‐LGI1 encephalitis. The weak positive correlation between baseline mRS and age at onset (*ρ* = 0.280) may reflect cumulative comorbidities in elderly patients, whereas the negative correlation between mRS and MoCA scores (*ρ* = −0.353) suggests a potential protective role of cognitive reserve in functional outcomes. After adjusting for sex, age, and disease duration, the associations of serum and CSF antibody titers with baseline mRS were significantly strengthened (partial *ρ* = 0.552 and 0.525, respectively), indicating that these covariates may have confounded the original associations: age‐related declines in neuroplasticity might amplify antibody‐mediated neurotoxicity, while prolonged disease duration could lead to fluctuations in antibody levels. Notably, the weak correlation between serum antibody titers and sex (*ρ* = 0.272) and the enhanced association between titers and mRS after sex adjustment imply that sex may modulate antibody effects through intricate mechanisms. These findings collectively underscore the necessity of accounting for demographic confounders when evaluating antibody‐clinical outcome relationships.

After adjusting for confounders (sex, age, disease duration), the initially weak correlation (*ρ* < 0.3) between parameters such as CSF white blood cell count and antibody titers became substantially strengthened (*ρ* = 0.414), suggesting that age‐related immune senescence and residual confounders may have obscured genuine biological associations. Furthermore, disease duration might act as a mediating variable by regulating the dynamic expression windows of inflammatory markers. These findings underscore the necessity of employing multivariate analyses to exclude confounding effects when interpreting weak correlations (*ρ* < 0.3) and to validate their reproducibility in independent cohorts.

In this cohort, 4 patients exhibited co‐occurrence of CSF anti‐LGI1‐Ab with additional autoantibodies, including anti‐metabotropic Glutamate Receptor 5 (mGluR5), anti‐Amphiphysin, and anti‐Titin antibody. One patient tested positive for both serum anti‐LGI1‐Ab and anti‐Yo antibody. The presence of multiple anti‐neuronal antibodies may trigger overlapping neurological syndromes due to synergistic pathogenic effects [25]. While LGI1‐AE is traditionally associated with a low tumor incidence [26], 7 patients (8.5%) in this study had underlying tumors, and 3 others showed positivity for paraneoplastic antibodies (anti‐amphiphysin, anti‐titin, anti‐Yo) despite normal tumor marker levels. These findings underscore the necessity for comprehensive tumor screening in all LGI1‐Ab‐positive patients.

In addition to CSF and serological indicators, commonly used auxiliary examinations include brain MRI, EEG, etc., but the sensitivity of brain MRI is limited (25%–50%). MRI can show abnormal signals in the unilateral or bilateral medial temporal lobe, or it may be normal, so PET‐CT examination can be performed when necessary [15, 27]. In this study, 49.4% of the cases had abnormal brain MRI, of which 14 cases had unilateral hippocampal and temporal lobe involvement, 23 cases had bilateral hippocampal and temporal lobe involvement, 6 cases were located in the BG, and 13 cases had bilateral or unilateral frontal, parietal, or occipital lobe slightly high‐density imaging. Therefore, these findings suggest that the diagnosis and treatment of this disease should be combined with other auxiliary examinations. Typically, only a small number of patients have MRI results that return to normal after immunotherapy, while most exhibit persistent hyperintensities or atrophy, which may be associated with ongoing neurological deficits [16].

Early‐stage FDG‐PET reveals increased metabolism in the medial temporal lobe and BG [15, 27]. The underlying mechanism involves intensive expression of LGI1 protein in the BD, hippocampus, and amygdala. LGI1‐Ab reduces neuronal membrane expression of potassium (Kv1.1) channels, leading to neuronal hyperexcitability and consequent metabolic hyperactivity. This pathophysiology explains the characteristic BG and temporal lobe hypermetabolism observed via FDG‐PET in LGI1‐AE patients. Selective BG hypermetabolism demonstrates a strong correlation with FBDS onset, and some cases may exhibit concurrent cerebellar and cortical hypermetabolism [16, 28]. In our cohort, 22 patients underwent complete FDG‐PET examination, with 16 (72.7%) showing abnormal findings: temporal lobe hypermetabolism in 13 cases, BG hypermetabolism in 5 cases, and hypometabolism in 13 cases affecting various regions (unilateral/bilateral cerebral cortex [frontal, parietal, or occipital lobes], basal ganglia, thalamus, cerebellum, and midbrain). Notably, among 36 FBDS patients, only 7 underwent FDG‐PET examination, with BG hypermetabolism detected in just 2 cases. This discrepancy might relate to the low FDG‐PET screening rate in this subgroup, necessitating further investigation for confirmation. The relatively specific FDG‐PET metabolic pattern observed in LGI1‐AE allows clinicians to recognize these characteristic features, providing critical diagnostic support for suspected autoimmune encephalitis cases with negative antibody results or unremarkable MRI findings.

Current clinical first‐line immunotherapy typically comprises corticosteroids, IVIg, and plasma exchange. Second‐line therapies such as rituximab, cyclophosphamide, and azathioprine are primarily reserved for patients exhibiting a suboptimal response to first‐line treatment or experiencing relapse [6]. In a longitudinal study by Rodriguez et al. involving 118 immunotherapy‐treated LGI1‐AE cases, corticosteroids demonstrated superior efficacy over IVIg in achieving acute‐phase clinical improvement [29]. In this study, glucocorticoid therapy was used in the acute stage; some patients were combined with IVIg, and (or) second‐line immunotherapy, and 15 cases were treated with plasma exchange, and their conditions improved to varying degrees before discharge, which was consistent with the results reported in previous studies [30].

This study has several limitations that warrant acknowledgment. First, as a multicenter retrospective study, inconsistencies in long‐term mRS follow‐up documentation were observed across participating centers. Furthermore, the predominant reliance on telephone‐based assessments may have compromised the precision of neurological function evaluations. Future research should implement prospective designs incorporating standardized outpatient follow‐up protocols (e.g., serial mRS combined with cognitive assessment) to enhance the reliability of long‐term prognostic data.

## 5 Conclusions

LGI1‐AE is a subtype of autoimmune limbic encephalitis characterized by clinical features including RPCI, BPS, seizures, and hyponatremia. LGI1‐AE is a subtype of autoimmune limbic encephalitis with common clinical features such as RPCI, BPS, seizures, and hyponatremia. Focal imaging and EEG abnormalities are commonly observed, particularly in the temporal lobe and basal ganglia. The positive correlation between CSF and serum LGI1‐Ab titers supports previous findings suggesting CSF LGI1‐Ab might originate peripherally. Further mechanistic studies on LGI1‐Ab production are required to develop precise targeted therapies. Acute‐stage serum and CSF LGI1‐Ab titers correlated with mRS scores, indicating higher titers correspond to stronger neurotoxicity, but no association was observed with disease recurrence.

Brain wave slowing may reflect underlying inflammatory processes, neuronal injury, or synaptic abnormalities in encephalitis. Further investigation is warranted to determine whether this feature could be a biomarker for early diagnosis or prognosis assessment. LGI1‐AE demonstrates favorable immunotherapy outcomes, with early, aggressive, and sustained combination immunotherapy associated with improved long‐term prognosis. The retrospective design, limited sample size, and heterogeneous follow‐up durations and criteria are key limitations. Given the above limitations, our results should be confirmed in prospective studies with larger cohorts.

## Author Contributions

L.S., J.T.Y., and Y.Z. contributed to the conception and design of the study. Y.Y., M.M.L., Y.Y.H., H.N.Z., Y.Z., M.Z., and H.T. contributed to the acquisition and analysis of data. Y.Z., Y.Y., R.Z.W., M.C., S.F.C., and K.L.C. contributed to drafting the text and preparing the figures.

## Ethics Statement

Approved by the Ethics Committee of Huashan Hospital Affiliated with Fudan University and the First Hospital of Jilin University, which agreed that this study met the conditions of exempting patients from informed consent.

## Consent

The authors have nothing to report.

## Conflicts of Interest

The authors declare no conflicts of interest.

## Supporting information


**Figure S1.** Comparison of α1‐band power values between the LGI1‐AE group and NC group.
**Figure S2.** Comparison of α2‐band power values between the LGI1‐AE group and NC group.
**Figure S3.** Comparison of β1‐band power values between the LGI1‐AE group and NC group.
**Figure S4.** Comparison of β2‐band power values between the LGI1‐AE group and NC group.

**Table S1.** Multivariable‐adjusted Correlations of mRS, Relapse, and LGI1 Antibody Levels with Clinical/Lab Markers in LGI1‐AE.

**Table S2.** Demographic data of two groups of subjects.

## Data Availability

Data are available upon reasonable request.
