# Supplementary figures and images for: Clinical, Electroencephalogram and Imaging Characteristics of Patients With Anti‐LGI1 Antibody Encephalitis: A Multicenter Cohort Study
Source: CNS Neurosci Ther. 2025 May 5;31(5):e70414. doi: 10.1111/cns.70414 (PMC12051031; doi:10.1111/cns.70414)

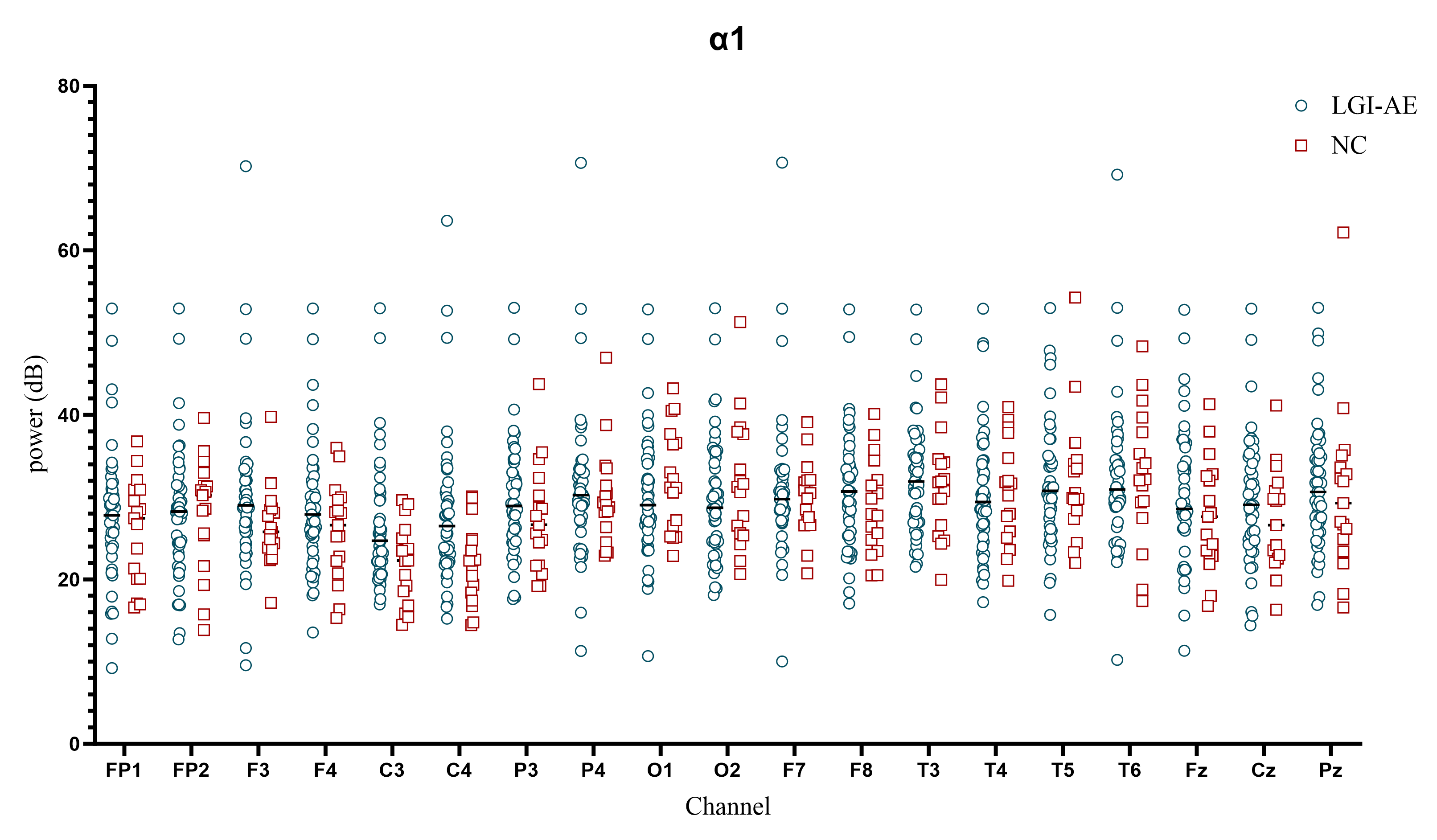

Supplement: Supplementary file 1 — Figure S1. Comparison of α1‐band power values between the LGI1‐AE group and NC group. Figure S2. Comparison of α2‐band power values between the LGI1‐AE group and NC group. Figure S3. Comparison of β1‐band power values between the LGI1‐AE group and NC group. Figure S4. Comparison of β2‐band power values between the LGI1‐AE group and NC group. [file CNS-31-e70414-s001.zip › Fig S1_2.tif]

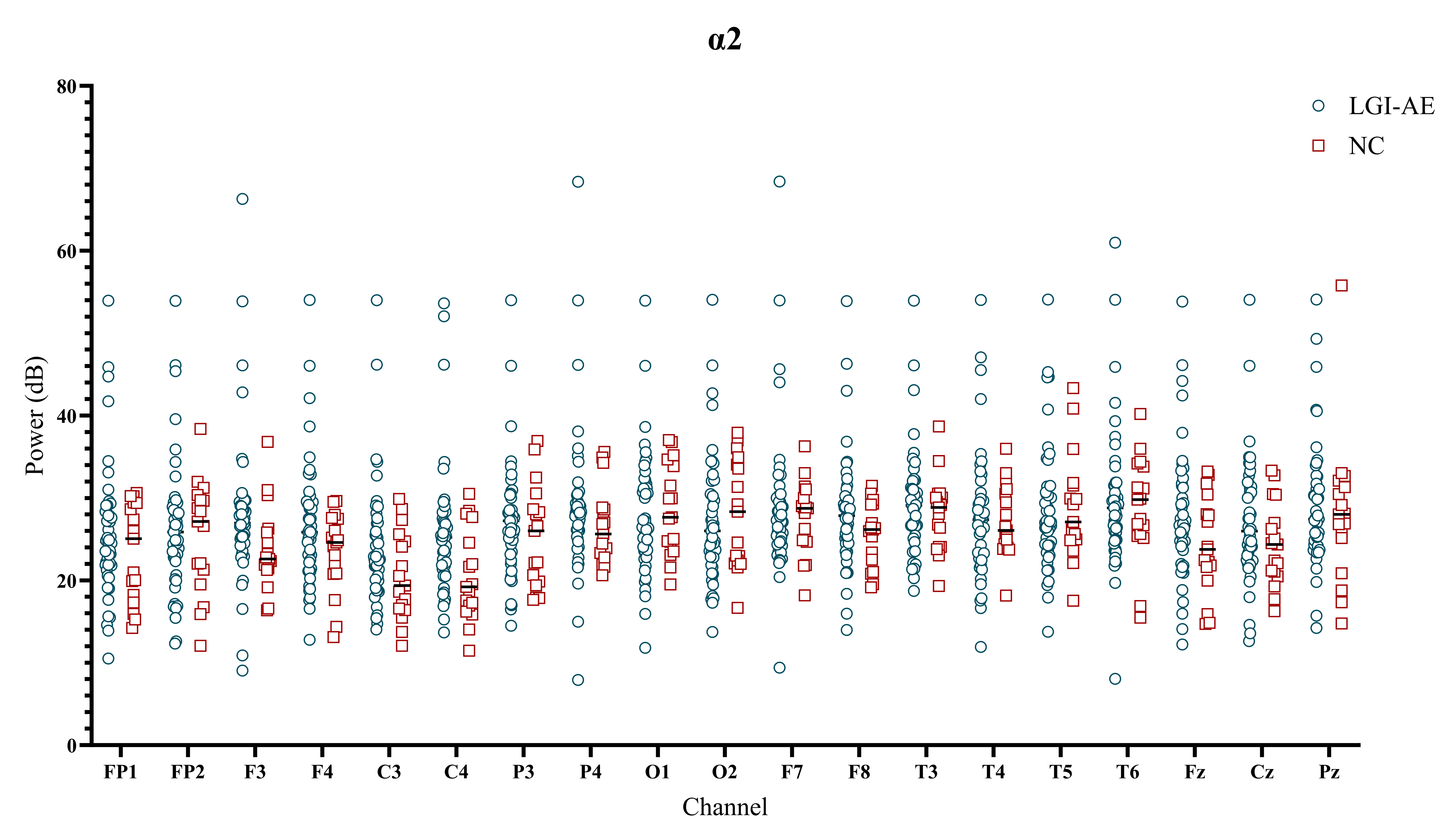

Supplement: Supplementary file 1 — Figure S1. Comparison of α1‐band power values between the LGI1‐AE group and NC group. Figure S2. Comparison of α2‐band power values between the LGI1‐AE group and NC group. Figure S3. Comparison of β1‐band power values between the LGI1‐AE group and NC group. Figure S4. Comparison of β2‐band power values between the LGI1‐AE group and NC group. [file CNS-31-e70414-s001.zip › Fig S2_2.tif]

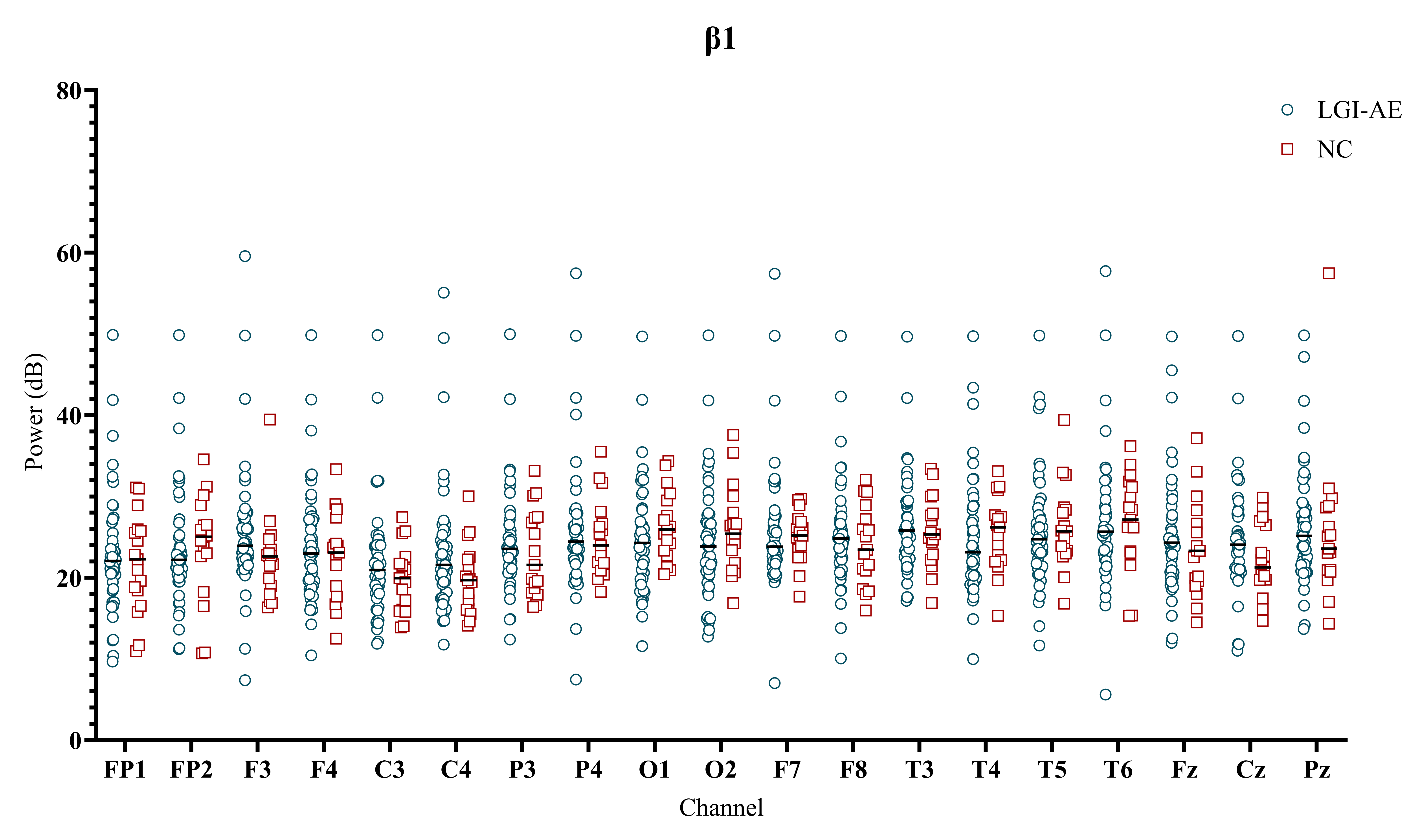

Supplement: Supplementary file 1 — Figure S1. Comparison of α1‐band power values between the LGI1‐AE group and NC group. Figure S2. Comparison of α2‐band power values between the LGI1‐AE group and NC group. Figure S3. Comparison of β1‐band power values between the LGI1‐AE group and NC group. Figure S4. Comparison of β2‐band power values between the LGI1‐AE group and NC group. [file CNS-31-e70414-s001.zip › Fig S3_2.tif]

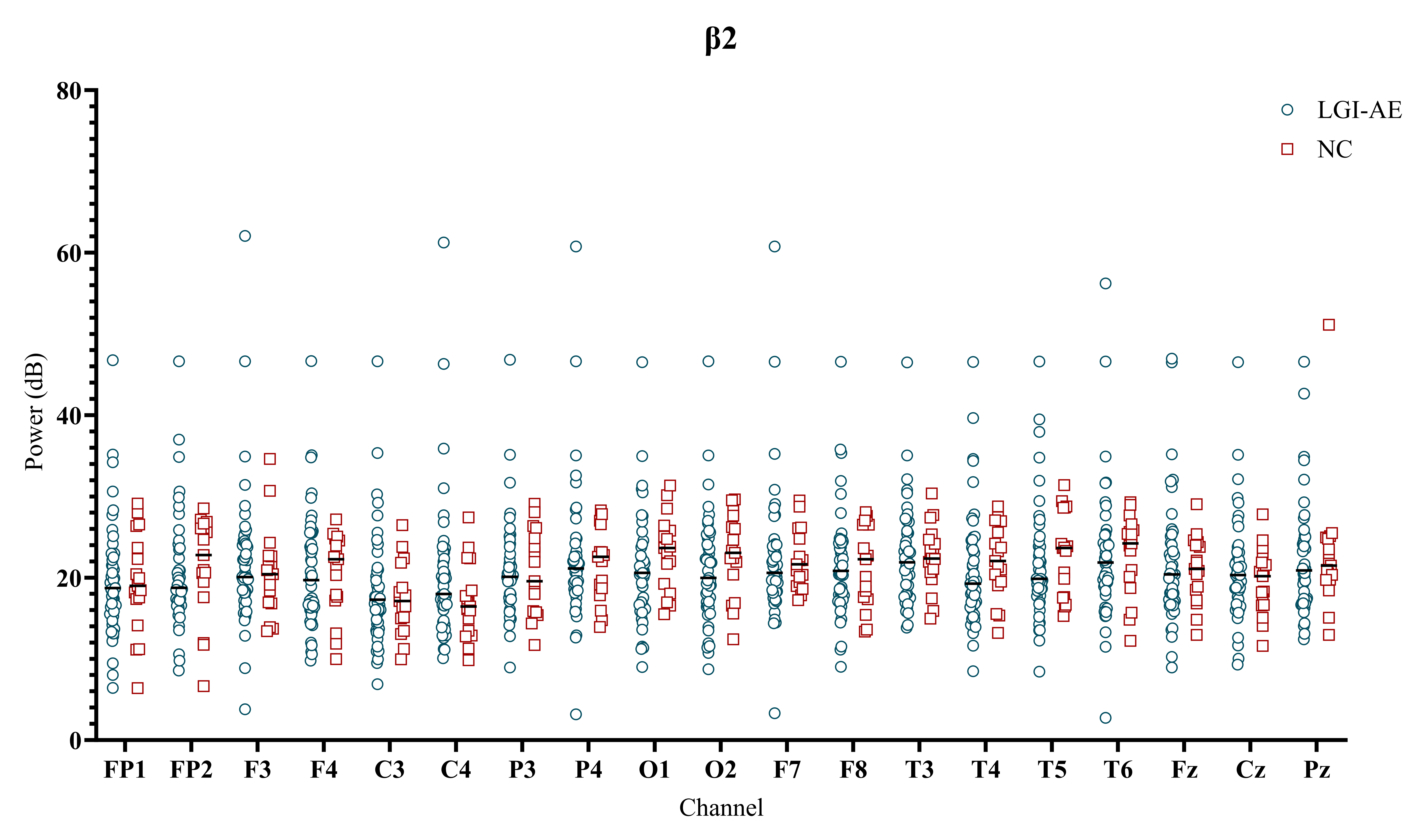

Supplement: Supplementary file 1 — Figure S1. Comparison of α1‐band power values between the LGI1‐AE group and NC group. Figure S2. Comparison of α2‐band power values between the LGI1‐AE group and NC group. Figure S3. Comparison of β1‐band power values between the LGI1‐AE group and NC group. Figure S4. Comparison of β2‐band power values between the LGI1‐AE group and NC group. [file CNS-31-e70414-s001.zip › Fig S4_2.tif]
