# Supplementary material for: Clinical, Electroencephalogram and Imaging Characteristics of Patients With Anti‐LGI1 Antibody Encephalitis: A Multicenter Cohort Study
Source: CNS Neurosci Ther. 2025 May 5;31(5):e70414. doi: 10.1111/cns.70414 (PMC12051031; doi:10.1111/cns.70414)
Supplement: Supplementary file 2 — Table S1. Multivariable‐adjusted Correlations of mRS, Relapse, and LGI1 Antibody Levels with Clinical/Lab Markers in LGI1‐AE. [file CNS-31-e70414-s002.docx]

| **Items** | **Baseline mRS** | **Recurrence** | **Serum anti-LGI1-Ab titer^1^** | **CSF anti-LGI1-Ab titer^1^** |
| --- | --- | --- | --- | --- |
| **Serum sodium** | -0.021 | 0.161 | -0.219 | 0.063 |
| **Intracranial pressure** | -0.439 | 0.115 | -0.273 | -0.188 |
| **CSF WBC** | 0.212 | 0.397 | **0.453*** | **0.414*** |
| **CSF Protein** | -0.173 | -0.128 | 0.144 | -0.268 |
| **CSF Chloride** | -0.079 | 0.127 | 0.152 | 0.380 |
| **MoCA** | 0.037 | 0.396 | 0.119 | 0.285 |
| **CSF anti-LGI1-Ab titer^1^** | **0.525**** | 0.094 | **0.561**** | - |
| **Serum anti-LGI1-Ab titer^1^** | **0.552**** | -0.004 | - | - |
| **Recurrence** | -0.051 | - | - | - |

Table S1 Multivariable-adjusted Correlations of mRS, Relapse, and LGI1 Antibody Levels with Clinical/Lab Markers in LGI1-AE

*: 0.01≤p<0.05; **: 0.001≤p<0.01; ***: p<0.001
