# Supplementary material for: Clinical, Electroencephalogram and Imaging Characteristics of Patients With Anti‐LGI1 Antibody Encephalitis: A Multicenter Cohort Study
Source: CNS Neurosci Ther. 2025 May 5;31(5):e70414. doi: 10.1111/cns.70414 (PMC12051031; doi:10.1111/cns.70414)
Supplement: Supplementary file 3 — Table S2. Demographic data of two groups of subjects. [file CNS-31-e70414-s003.docx]

| **Items** | **LGI1-AE (n=47)** | **NC (n=17)** | **P value** |
| --- | --- | --- | --- |
| **Female (n%)** | 24 (51.1%) | 9 (50%) | 0.894 |
| **Age [year，mean(SD)]** | 57.45 (11.33) | 62.24 (7.41) | 0.111 |
| **MMSE [mean(SD)]** | - | 28.29 (1.05) | - |
| **MoCA [mean(SD)]** | - | 24.89 (2.09) | - |

Table S2 Demographic data of two groups of subjects

Abbreviations: LGI1-AE = LGI1 antibody-associated autoimmune encephalitis; NC = normal control; MMSE = mini-mental state examination; MoCA = montreal cognitive assessment.
